# The Influence of Reading Texts on L2 Reading-to-Write Argumentative Writing

**DOI:** 10.3389/fpsyg.2021.655601

**Published:** 2021-03-19

**Authors:** Jingjing Qin, Yingliang Liu

**Affiliations:** ^1^Department of Language Studies, College of Humanities and Social Sciences, Zayed University, Abu Dhabi, United Arab Emirates; ^2^Department of English, School of Foreign Languages, Wuhan University of Technology, Wuhan, China

**Keywords:** reading-to-write tasks, argumentative writing, Toulmin model, reading texts, L2 writers

## Abstract

Reading-to-write is an essential skill in academic writing, and reading-writing tasks have been widely adopted in standardized English tests. Much more recent literature on integrated reading-writing tasks has focused on writers’ use of source texts or the validity of integrated writing assessment, while little is known about whether the nature of the types of reading texts has any bearing on integrated reading-writing tasks. This study examines whether the types of reading texts (i.e., similar views or opposing views on a debatable issue) have any influence on second language (L2) students’ argumentative writing in terms of the use of argument elements and its overall quality. Fifty-four Chinese second-year university students majoring in English language teaching were asked to write an argumentative essay after reading texts with either similar views or opposing views. Results show that students reading texts with opposing viewpoints presented more data and higher overall quality of argumentative essays than students reading texts with similar viewpoints, although the latter group presented more counterargument data. Pedagogical implications on teaching argumentative writing are discussed.

## Introduction

Reading-to-write tasks have been a common assessment tool in standardized English tests, such as the Test of English as a Foreign Language (TOEFL), the International English Language Testing System (IELTS), and the Canadian Academic English Language (CAEL) (Weigle and Parker, 2012). Compared to independent writing tasks, reading-to-write tasks are more complex as they require writers to check their understanding of the tasks and interact with the source texts during the whole process (Plakans, 2008, 2010). Writing from multiple sources poses additional challenges in that writers need to choose the appropriate organizational structure in order to integrate information from different sources (Wiley and Voss, 1999; Mateo et al., 2011).

Given their prevalence in academic contexts, the complexity of reading-to-write tasks has attracted much research interest both in L1 and L2 contexts. The intricacy of these reading-to-write tasks resides in the complex processes involved in both reading and writing. Related studies on writing from sources in the discipline of history have shown that students are more likely to develop complex historical reasoning when presented with multiple documents, in particular when the multiple documents present oppositional information (Rouet et al., 1996, 1997). It follows that the combination of multiple source texts prior to assigning an argumentative writing task on the same topic would be expected to prompt learners’ more active processing mechanisms in a learning context. However, writing from sources is an academic literacy task likely to be encountered by students across disciplines, as mentioned earlier, and up to date, relatively scarce research has been done yet to investigate how reading texts influence writing in a reading-to-write task in L2 academic contexts. The answer to this question can help shed light on this aspect of the complex, multifaceted integrated task.

According to the presumed role of source texts in the writing task commonly used in tests, Guo (2011) has classified three types of reading-to-write tasks. The first type requires using reading texts as the main source of information for the written products as in summaries; the second type are situation-based writing tasks, which are more casual tasks including emails or letters in response to prompts such as conversations or written notes; the third type is thematically related writing tasks, often argumentative tasks, which ask writers to present their own viewpoint on a controversial issue, supported by information from reading texts as well as writers’ own experience and knowledge. Apparently, the third type of writing tasks is the most complex as it involves more in-depth analysis and integration of information from the reading texts. Therefore, this type of integrated writing task has attracted more attention (Shi, 2004; Weigle and Parker, 2012; Plakans and Gebril, 2013; Gebril and Plakans, 2015). For example, with a think-aloud and a questionnaire, Plakans and Gebril (2013) found that L2 university students relied on source texts to gather ideas about the topic or evidence and to follow the organization. Examining the use of source texts from the perspective of metacognitive strategies, Golparvar and Khafi (2021) suggested that planning and evaluation were adopted by students with a higher level of self-efficacy in their summary writing. These metacognitive strategies, though having no direct impact on students’ writing performance, enabled student writers to consciously select information, organize ideas, paraphrase and quote.

In terms of argumentative writing, Wiley and Voss (1999) have shown that argumentative writing from several sources prompted a significantly deeper understanding of source texts than writing from a single source. Compared to narrative writing, participants who wrote arguments produced essays with more integrated and transformed ideas with causal thinking. Moreover, Anmarkrud et al. (2014) found that students’ argumentative writing about an unfamiliar scientific issue was related to their strategic processing during reading conflicting documents on the issue, and their argumentative reasoning was positively related to evaluating, monitoring and linking documents. As seen, so far, relatively little research has investigated the impact of reading texts on L2 argumentative writing. Therefore, the present study is intended to fill in the gap by investigating argumentative writing tasks by L2 university writers, who are asked to support their own stances on a debatable topic after reading two argumentative texts with either similar or opposing views on the same topic.

Of particular relevance to the study presented here are the frameworks used to analyze argumentative texts. Having been shown as an effective way of evaluating the effectiveness of argumentation, Toulmin’s argument model (1958, 2003) has been extensively adopted in research on both L1 and L2 argumentative writing (Hitchcock, 2005; Voss, 2005; Davies, 2008; Qin and Karabacak, 2010; Liu and Stapleton, 2014; Stapleton and Wu, 2015; Abdollahzadeh et al., 2017). According to this model, effective argumentation presents six elements: claim, data, and warrant, qualifiers, backing, and rebuttals; the first three are the main elements of every argument and the latter three are the second-level elements, the presence of which are affected by the circumstances of the argument. The present study adapted Toulmin model of argument structure (1958, 2003), and only analyzed the following elements, namely, claim, data, counterargument claim, counterargument data, rebuttal claim, and rebuttal data, as supported by a number of previous studies that these are the more commonly found elements in L2 students’ writing (Qin and Karabacak, 2010; Liu and Stapleton, 2014; Abdollahzadeh et al., 2017).

To further investigate the relationship between the types of reading texts and the writing quality of L2 students, this study examines whether the different reading text types (i.e., with similar or opposing views on a debatable issue) have any influence on students’ argumentative writing. Specifically, the study will address the following research questions:

1. How the types of reading texts influence argumentative elements in argumentative papers written by L2 university students, based on the adapted Toulmin model of argument (1958, 2003)?
2. How the types of reading texts influence the overall quality of argumentative papers written by L2 university students?

## Methods

### Participants

The participants of the study were based on a convenience sample, a total of 54 second-year Chinese university students majoring in English (45 female and 9 male) taking an English academic writing course with the second author. Despite some inherent drawbacks in the convenience sample, it is still a commonly used sampling strategy in second language classroom-based research for its practicality and viability (Dornyei, 2007; Creswell and Creswell, 2018). Students were around the same age, 18–21 years old, and they had learned English for 10 years by the time of data collection. The data were collected when these students took an academic writing class with the second author, and they had completed an introductory writing course for one semester prior to this one. Thus, they had already practiced writing different types of genres, including narrative, expository, and argumentative essays. In preparation for the writing component of the Test for English Majors, Band 4 (TEM-4), a national English proficiency test, students completed two timed argumentative essays during the semester, which were rated by two experienced writing instructors based on the TEM-4 writing rubric. For data collection, the students were randomly divided into two groups of 27 students, and there was no significant difference in the mean scores of the two-timed essays by the two groups.

### Writing Task

As mentioned above, integrated reading-to-write tasks with the genre of argumentative writing are the most commonly assigned writing tasks in university academic studies, and thus often adopted in internationally standardized tests. The main purpose of this study was to examine the effect of reading text types on argumentative writing. To this end, the participants were asked to write an argumentative paper based on two reading texts; one group were randomly assigned to two texts with similar views, and the other group to two texts with opposing views. The writing topic had been carefully chosen, namely, whether the future status of English as the global language will be assured or not. This topic was selected based on the rationale that the participants would have sufficient background knowledge as well as a strong interest in writing about because of their major in English. The reading levels and the length of the texts were fully controlled. The two texts with similar views are “Brazil Considers Linguistic Barricade” (from *Christian Science Monitor*, 728 words) and “English in Decline as a First Language, Study Says” (from *National Geographic News*, 842 words). The two texts with opposing views are “The Triumph of English as the World’s Language” (from *Bangor Daily News*, 884 words) and “English in Decline as a First Language, Study Says”. Students were required to read the two texts in 25 min and write an argumentative paper (350 to 500 words in length) in 45 min. The readability levels of the three texts as determined by the Flesch-Kincaid grade levels were between the 10th and the 12th grade, deemed appropriate for the reading levels of these participants. Students were asked to support their stance with information provided in the two texts. In addition, they could use any other information from their personal knowledge or experience to support their views.

### Analysis

Three essays were discarded because they were either too short (less than 250 words) or incomplete, and thus 51 essays were included in the final analysis. To examine the details of the argument structure, specific elements of the argument were analyzed based on the adapted Toulmin model of argument structure (1958, 2003), namely, claim, data, counterargument claim, counterargument data, rebuttal claim, and rebuttal data. The definition and example of each element are presented in Table 1. The two authors first independently coded 10 papers randomly selected from the data and the inter-rater reliability for coding the six Toulmin elements, claim, data, counterargument claim, counterargument data, rebuttal claim, and rebuttal data in terms of Cohen’s Kappa was 0.80, 0.85, 0.97, 0.96, 0.96, and 0.97, respectively, and overall inter-rater reliability was 0.86. The second author then coded the rest of the data, and the first author double-checked the coding and reached agreement in case of discrepancies. To evaluate the quality of the students’ argumentative papers, a 5-scale scoring rubric (see Supplementary Appendix A) was adopted from Qin and Karabacak (2010). Based on the rubrics used by McCann (1989) and Nussbaum and Kardash (2005), this scoring rubric provides a clear description of the overall effectiveness of the argument (including possible counterarguments and rebuttals), organization, and language use. Each paper was independently rated by two trained raters with experience in teaching L2 writing, and the interrater reliability coefficient alpha was0.86. The average of the two scores was taken as the score of the paper. In case of disagreement, that is, when the two scores were more than one point apart, discussion and negotiation were made between the two raters until agreement was reached. To compare the argument elements and overall quality of the essays by the two groups, independent-samples *t*-tests were performed, and the results are presented in Tables 2, 3.

**TABLE 1:** Definitions and examples of six Toulmin elements (Ramage and Bean, 1999).

Toulmin elements	Definitions and examples
Claim	**Definition:** An assertion in response to a contentious topic or problem. **Example:** As a language that has acted as a global language for years, English certainly will continue its role in the future.
Data	**Definition:** Evidence to support a claim. It can take various forms, such as facts, statistics, research studies, anecdotes, analogies, expert opinions, definitions and logical explanations. **Example:** 1. John Adams, the second president of the United States, once predicted that English would be global language and everybody in the world would accept it (expert opinions). 2. According to one report, the Politecnico di Milano of Italian University, will teach in English in all of its courses from 2014 (fact).
Counter-argument claim	**Definition:** The possible opposing views that challenge the validity of a writer’s claim. **Example:** Thus some people doubt the situation of English as a first language in the future.
Counter-argument data	**Definition:** Evidence similar to “Data” (above) to support a counterargument claim **Example:** 1. On the other hand, with more and more people join the world market, they have more choices to decide which language they learn (fact). 2. According to the facts, the population grew up speaking English as their first language is expected to be 5 percent in 2050 (statistics).
Rebuttal claim	**Definition:** Statements in which the writer responds to a counterargument claim **Example:** In the future there will be more and more people speak English as their second language.
Rebuttal data	**Definition:** Evidence to support a rebuttal claim by pointing out the possible weakness in the counterargument claim, data, or warrant, such as logical fallacies, insufficient support, invalid assumptions, and immortal values. **Example:** (But I think there is no contradiction between the mother language and the second language.) We need to learn English to adopt the society and we also need to learn Chinese to develop our own culture. We can combine them with each other.

*Examples are chosen from the data of the current study.*

**TABLE 2:** Use of Toulmin elements by two groups.

	Texts with similar views (*N* = 28)		Texts with opposing views (*N* = 23)	
	Mean	SD	Mean	SD
Claim	3.75	1.62	4.09	1.51
Data	5.93	2.39	8.61*	3.58
Counter-argument claim	0.57	0.50	0.52	0.59
Counter-argument data	1.46*	1.23	0.57	1.04
Rebuttal claim	0.25	0.44	0.30	0.47
Rebuttal data	0.50	1.00	0.35	0.78

*indicates *p* < 0.05.

**TABLE 3:** Quality of essays by two groups.

	Texts with similar views (*N* = 28)		Texts with opposing views (*N* = 23)	
	Mean	SD	Mean	SD
Scores	3.18	0.61	3.57*	0.51

*indicates *p* < 0.05.

## Results

### Argument Elements in the Essays

To address the first research question on the argument elements used in the argumentative papers, all 51 essays were coded following the adapted Toulmin’s (1958, 2003) argument elements, including claim, data, counter-argument claim, counter-argument data, rebuttal claim and rebuttal data. The result is presented in Table 2. The hypothesis of the study is that students who have read texts with opposing views would include more data and counterargument data in their argumentative essays than those having read texts with similar views because the former group was exposed to both sides of the view and more data to support either side. As shown in Table 2, students reading texts with opposing views presented more data in their essays (*M* = 8.61, *p* < 0.05); surprisingly, students reading texts with similar views presented more counterargument data (*M* = 1.46, *p* < 0.05).

### Overall Essay Quality

To answer the second research question on the writing quality, all essays were rated and the quality of the essays by the two groups is presented in Table 3. The results show that the group reading texts with opposition views (*M* = 3.57, *p* < 0.05) perform significantly better in their papers than the group reading texts with similar views (*M* = 3.18). This result concurs with the hypothesis that students who read texts with opposing views were more likely to be exposed to different views of a controversial issue and thus, engaging in more in-depth argumentation discussion.

## Discussion and Implications

This study examined how reading texts influenced L2 argumentative writing in terms of its argument elements and the overall quality. The overall results showed that the types of reading texts had an impact on the argument elements and the quality of essays.

A closer look at the positions held by the two groups of students may reveal some interesting results. Table 4 shows the positions held by the two groups of students in their essays. As shown in Table 4, almost half of the students reading texts with similar views (13 out of 28) supported the position that the future status of English as the global language will not be assured, which is consistent with the positions in the two reading texts. Eight students reading texts with similar views held the opposite position, which disagrees with that of the two reading texts. The rest seven students reading texts with similar views never answered the question in their entire essays directly and their argument went astray to other issues such as the necessity of learning English, the importance to maintain the mother tongue, and the trend of multilingualism. In contrast, a majority of students (78.26%) reading texts with opposing views chose to support the position that the future status of English as the global language would be assured. Only four students held the opposite position, and one student had an unclear position, that is, the claim does not address the main issue directly. It is interesting to note that students reading texts with similar views that *the future status of English as the global language will not be assured* were shown with the obvious tendency to choose the same position as the reading texts, although these students majoring in English teaching, presumably, were more likely to oppose this view presented in the two reading texts by arguing for the opposite. This may suggest the strong influence of reading texts on the writing product in terms of content. In other words, students may simply follow the position from reading texts even if it might be opposite to their initial ideas. It should be noted that neither of the reading texts with similar views addresses the counterargument that *the future status of English as the global language will be assured*. This finding was consistent with Plakans and Gebril (2012) who examined L2 University students’ use of source texts through think-aloud and a questionnaire and found that the students gained ideas about the topic and adopted organizational patterns from reading texts.

**TABLE 4:** Positions supported in essays by two groups.

Positions	Texts with similar views (*N* = 28)	Texts with opposing views (*N* = 23)
Status of English will be assured	8 (28.57%)	18 (78.26%)
Status of English will not be assured	13 (46.43%)	4 (17.39%)
Unclear	7 (25.00%)	1 (4.35%)

It was also found that students reading texts with opposing views included more data. Inclusion of more data in the essays by this group suggests that they might tend to have a deeper understanding of the controversial issue and were engaged with the evaluation of both sides of the issue (Anmarkrud et al., 2014). They were also more likely to clearly show the position at the beginning of the essay and support it with more data. Students reading texts with similar views, nevertheless, were more prone to agree with the positions in the reading texts, and some of them were even distracted by the information in the texts while forgetting to address the main issue in the writing task. It turns out that most of the counterargument data were found in the essays with unclear positions on the issue. In these essays, the student writers make claims on some related issues covered in the reading texts instead of the main issue, namely, *the future status of English as a global language*. For example, “Perhaps those shopkeepers think English names are popular, but I think using English names instead of native ones is not a good idea.” Neither was the counterargument data related to the main issue, for example, “Brazil is like this, and abuse of English expressions happened in this country. Many shops use English names instead of Portuguese ones.” Compared to the use of claim and data, very few counterargument claim, counterargument data, rebuttal claim, rebuttal data were included in the essays by students in both groups, which is consistent with previous findings on EFL argumentative writing (Qin and Karabacak, 2010; Liu and Stapleton, 2014; Abdollahzadeh et al., 2017). Despite the vital role counterarguments play in argument (Toulmin’s, 2003), a majority of the EFL students in the current study may not be aware of the effectiveness of the four second-level elements in the quality of the argument.

Students reading texts with opposing views outperformed students reading the text with similar views in the overall quality of the argumentative essays they produced, which concurs with findings of previous studies that argumentative writings from sources with opposite viewpoints are involved in deeper reasoning (Rouet et al., 1996, 1997). Inclusion of more data can strengthen the argument, hence, improving the overall quality of the essay. Counterargument data, though an important element in Toulmin’s model (2005), may not improve the effectiveness of the argument if rebuttal claim and rebuttal data are not included or the claim fail to present a clear position on the main issue. Since rebuttal claim and rebuttal data were scarcely found in essays by both groups, more counterargument data in the essays by students reading texts with similar views led to more weakening of the essays’ overall quality. This result also confirms the finding that compared to data, counterargument data is weakly correlated to the overall writing quality (Abdollahzadeh et al., 2017).

The difference in the quality of the essay can be further explained by the position supported in the essays. All essays with ambiguous positions were scored 2 or 3 out of 5. Among the eight essays holding ambiguous positions, seven of them are from the group reading texts with similar views, which suggests that exposure to texts of conflicting views may encourage students to understand the different sides of the issue and thus, are more likely to take sides with one. Some potential pedagogical implications for teaching L2 argumentative writing can be drawn from the results of the study. Firstly, exposure to texts with opposing views can lead to higher-quality argument essays, as students are more likely to be engaged in a critical evaluation of both sides of a controversial issue. Application of this type of integrated argumentative writing task should be encouraged in college writing classes. By providing multiple reading texts with different views on the same issue, the teacher can guide students to evaluate the strengths and weaknesses of the argument in the texts. Secondly, the findings suggest that most L2 university students share a similar understanding of the argument where they present data and claims, which is also a preferred argument structure suggested in most of the writing textbooks. However, the other four secondary-level Toulmin elements (counterargument claim, counterargument data, rebuttal claim and rebuttal data) are seldom presented despite the types of reading texts. Up till now, a great deal of research has documented L2 university students’ challenges with the understanding of the argumentative genre, and according to Wei et al. (2020), L2 writers’ perception of writing challenges is a strong contributor to this outcome, and even advanced L2 writers reverted to L1 rhetorical patterns when encountering difficulties in L2 argument structure. Since the four secondary-level Toulmin elements (counterargument claim, counterargument data, rebuttal claim, and rebuttal data) are often non-existent in the students’ L1 rhetoric conventions, as suggested by a review of contemporary Chinese writing textbooks (Kirkpatrick and Xu, 2012), the positive L1-to-L2 transfer is very unlikely to occur. Thus, it is suggested that Toulmin model, proved to be an effective model of argument, can be introduced explicitly to students to enhance their awareness of opposing views and the necessity to refute counterarguments when developing an argument (Liu and Stapleton, 2014).

The present study is limited in two ways, which can suggest directions for future research. Firstly, the small sample sizes of the writing samples limited the generalizations of the findings to the EFL context. Replication of the study with a larger number of participants can increase the generalizability. Secondly, although the study has shown that the type of reading text has some effect on the inclusion of argument elements, it remains unknown how students use information from the reading texts to support their argument. Future research can investigate students’ strategies of using sources (Weigle and Parker, 2012; Plakans and Gebril, 2013) and how the sources are integrated in the argument structures.

## Data Availability Statement

The raw data supporting the conclusions of this article will be made available by the authors, without undue reservation, to any qualified researcher.

## Ethics Statement

The studies involving human participants were reviewed and approved by the Research Ethics Committee, Zayed University. The participants provided their written informed consent to participate in this study.

## Author Contributions

JQ conceived of the initial idea, designed the study, and drafted the manuscript. YL collected and analyzed the data and drafted the manuscript. JQ and YL revised and proofread the manuscript. Both authors contributed to the article and approved the submitted version.

## Conflict of Interest

The authors declare that the research was conducted in the absence of any commercial or financial relationships that could be construed as a potential conflict of interest.
