# Supplementary material for: The Influence of Reading Texts on L2 Reading-to-Write Argumentative Writing
Source: Front Psychol. 2021 Mar 19;12:655601. doi: 10.3389/fpsyg.2021.655601 (PMC8017206; doi:10.3389/fpsyg.2021.655601)
Supplement: Supplementary file 2 [file Table_1.DOCX]

Supplementary Material

# Appendix A: A holistic scoring rubric for English argumentative papers

Scale 5: An excellent persuasive argument. The paper states a clear point of view and gives good and sufficient reasons to support it. The reasons are clearly explained and well-elaborated by using convincing information/ examples. The paper may present reasonable opposing view(s) and also refute the opposing view(s) appropriately, though they are not required. The paper is well-organized and sequenced. It demonstrates effective word choice and contains few or no grammar/mechanical errors.

Scale 4: A reasonably good and persuasive argument. The paper states a reasonably clear point of view and gives generally plausible reasons to support it. The reasons are explained and elaborated to some extent, though not enough. There may be one or two inconsistencies or pieces of irrelevant information. The paper may present some opposing point of view(s), but may fail to refute them or the refutation may be ineffective. The paper is generally well-organized and flows well, and shows evidence of effective word choice overall. There might be some grammar/mechanical errors, but they do not impede overall communication.

Scale 3: A clearly recognizable argument but limited in effectiveness. The paper states a point of view and gives one or two good reasons to support it. The reasons are not explained or supported in a fully coherent way. The reasons may be of limited plausibility and some inconsistencies exist. The organization is not well-developed, and ideas could be better sequenced. There might be some major problems in word choice; a noticeable number of grammar/mechanical errors occur.

Scale 2: A minimally acceptable argument paper, though not persuasive. The paper states a point of view but only one good reason is provided to support the point of view; or the reasons given are unrelated to or inconsistent with the point of view; or the reasons are incoherent. The organization is weak and ideas are not sequenced well. The paper demonstrates limited control of written language. There are numerous word choice, grammar and mechanical errors, and communication is impeded by these errors.

Scale 1: An ineffective argument with major gaps in reasoning. The paper states some sort of a point of view, but it is vague or general. No reasons are provided for the point of view; or the reasons given are unrelated to or inconsistent with the point of view. Most of the content of the paper is not relevant to the task. The paper is not properly organized, and it just contains piece meals of list of phrases or sentences with no coherence. There are so many word choice, grammar and mechanical errors that communication is severely impeded by these errors.
